# snoRNAs: functions and mechanisms in biological processes, and roles in tumor pathophysiology

**DOI:** 10.1038/s41420-022-01056-8

**Published:** 2022-05-12

**Authors:** Zheng-hao Huang, Yu-ping Du, Jing-tao Wen, Bing-feng Lu, Yang Zhao

**Affiliations:** Department of Obstetrics and Gynecology, Department of Gynecologic Oncology Research Office, Guangdong Provincial Key Laboratory of Major Obstetric Diseases, The Third Affiliated Hospital of Guangzhou Medical University, Guangzhou, China

**Keywords:** Tumour biomarkers, Cancer epigenetics

## Abstract

Small nucleolar RNAs (snoRNAs), a type of non-coding RNA, are widely present in the nucleoli of eukaryotic cells and play an important role in rRNA modification. With the recent increase in research on snoRNAs, new evidence has emerged indicating that snoRNAs also participate in tRNA and mRNA modification. Studies suggest that numerous snoRNAs, including tumor-promoting and tumor-suppressing snoRNAs, are not only dysregulated in tumors but also show associations with clinical prognosis. In this review, we summarize the reported functions of snoRNAs and the possible mechanisms underlying their role in tumorigenesis and cancer development to guide the snoRNA-based clinical diagnosis and treatment of cancer in the future.

## FACTS

- SnoRNAs can be mainly divided into three types: H/ACA box snoRNAs, C/D box snoRNAs, and scaRNAs.
- SnoRNAs are related to the modification of RNAs, including 2′-O-methylation and pseudouridylation of rRNAs and ac4C of 18 S rRNA. SnoRNAs can also regulate alternative splicing and have a function like miRNAs.
- SnoRNAs take part in the occurrence and development of cancers.

## OPEN QUESTIONS

- How are snoRNAs produced and what are their functions?
- How do snoRNAs take part in biological processes?
- How do snoRNAs take part in tumorigenesis and cancer development?

## Introduction

**Fig. 1:**
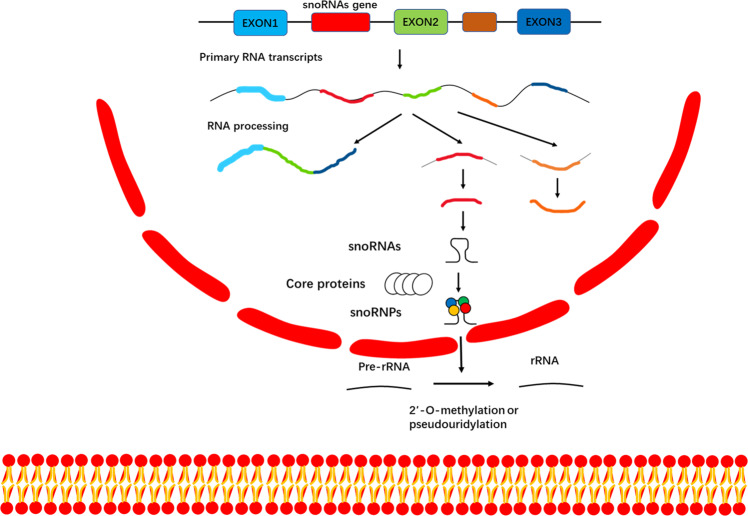
The biosynthesis of snoRNAs and snoRNPs. Originate from the nucleolus, snoRNAs are mainly encoded in the intron region of the gene transcribed by RNA polymerase II. SnoRNAs form functional snoRNPs through binding to core proteins. SnoRNAs stabilize the structure of rRNA through modifying rRNA with 2 ‘- O-methylation and pseudouridylation.

Small nucleolar RNAs (snoRNAs) are small non-coding RNAs widely present in the nucleoli of eukaryotic cells and have a length of 60–300 nt [1]. snoRNAs are mainly encoded by intronic regions of both protein coding and non-protein coding genes [2]. Normally, snoRNAs can be mainly classified into three groups: H/ACA box snoRNAs, C/D box snoRNAs, and small cajal RNAs (scaRNAs) [3]. The former two types of snoRNAs participate in the processing of ribosomal RNA (rRNA) by adding 2′-O-methylation and pseudouridylation modifications to rRNA molecules, respectively. However, a type of snoRNAs are located at Cajal bodies (CBs), so they are called scaRNAs. They also follow C/D-H/ACA classification, but some scaRNAs contain both C/D and H/ACA structures [4]. C/D box snoRNAs bind to four essential proteins—Nop1p, Nop56p, Nop58p, and Snu13p—to generate functional small nucleolar ribonucleoproteins (snoRNPs). Likewise, H/ACA box snoRNAs form functional snoRNPs by binding to Cbf5p, Gar1p, Nhp2p, and Nop10p [5] (Fig. 1).

The length of eukaryotic C/D box snoRNAs usually ranges from 70 to 120 nt. These snoRNAs contain two conserved sequences: the C box and the D box. The C box consists of the nucleotides RUGAUGA, which are located at the 5′-end of the snoRNA molecule. In contrast, the D box is located at the 3′-end and consists of the nucleotides CUGA [6]. Together, these elements depend on the base-pairing to fold into a structure called a kink-turn. This structure is recognized by Snu13p, which then recruits Nop1p (also called fibrillarin [FBL]), Nop58p, and Nop56p for 2′-O-methylation modification [5, 7].

**Fig. 2:**
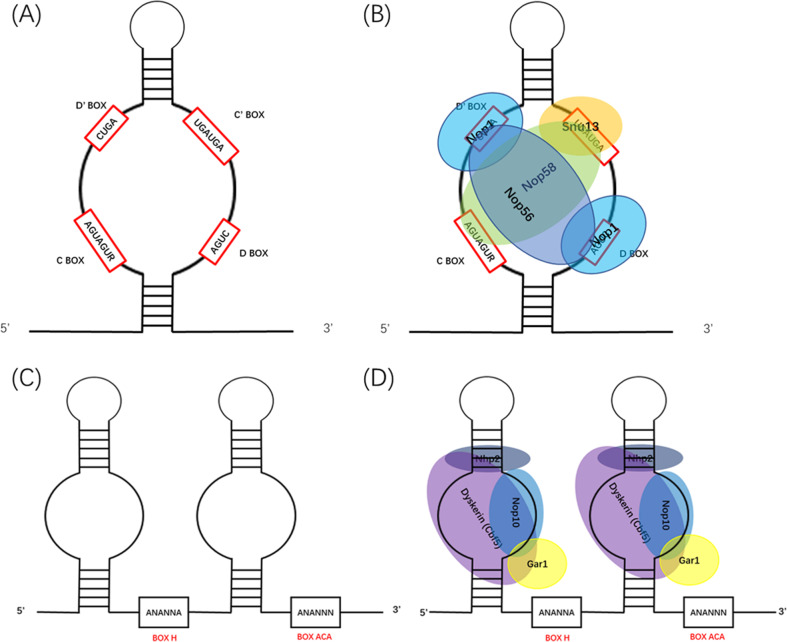
Structures of snoRNAs and snoRNPs. The structure of C/D box snoRNAs **A**, C/D box snoRNPs **B**, H/ACA box snoRNAs **C**, and H/ACA box snoRNPs **D**.

H/ACA snoRNAs are usually 60–75 nt in length and contain the region called the pseudouridylation pockets wherein uridine residues on the substrate RNA are isomerized [8]. H/ACA box snoRNPs bind to Cbf5p, Nop10p, Gar1p, and Nhp2p, among which Cbf5p acts as the catalytic protein involved in pseudouridylation [9]. Eukaryotic H/ACA box snoRNAs contain two conserved sequences: the H box and the ACA box, which are located downstream of the first and second hairpin, respectively [10] (Fig. 2).

Besides, some snoRNAs have been found that they have no apparent complementarity with rRNAs at known modified positions and they are called orphan snoRNAs. These indicated that snoRNAs have more function other than 2′-O-methylation and pseudouridylation of rRNAs. Kishore et al. indicated that HBII-52/SNORD115 had no complementarity with known modified positions of canonical snoRNAs. They found that HBII-52 bound to exon Vb of the 5-HT2C receptor and regulated alternative splicing [11, 12]. Another study found that orphan snoRNA SNORA73 inhibits PARP1 auto-PARylation to affect cancer genome stability by forming a small nucleolar ribonucleoprotein with PARP1 and DKC1/NHP2 [13].

SnoRNAs are also reported that they play a significant role in several tumors, such as lung cancer, gastric cancer. colorectal cancer, breast cancer, and so on. This review focuses on the functions of snoRNAs and the possible regulatory mechanisms underlying their role in biological processes, as well as their involvement in cancer pathophysiology.

## Possible molecular mechanisms underlying the role of snoRNAs in biological processes

**Fig. 3:**
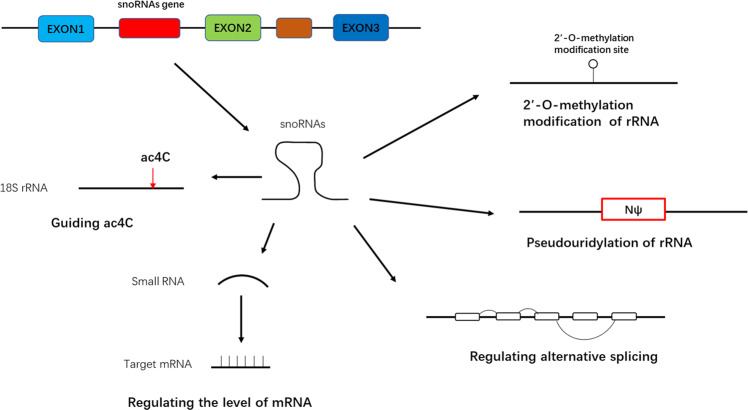
Molecular mechanisms of snoRNAs in biological processes. The two common mechanisms of snoRNAs contain 2′-O-methylation and pseudouridylation of rRNAs. It is reported that snoRNAs also can regulate alternative splicing, guide N4-acetylcytidine and regulate the level of mRNA like a miRNA.

The common actions of snoRNAs include the 2′-O-methylation and pseudouridylation of rRNAs [14]. In recent years, there has been increasing research on snoRNAs, and several studies have confirmed that snoRNAs can also regulate cell physiology by guiding N4-acetylcytidine (ac4C) modifications, regulating alternative splicing (AS), and performing miRNA-like functions (Fig. 3).

### 2′-O-methylation

In 1981, Langberg et al. first detected methyltransferase activity in extracts from HeLa cells [15]. In 2000, it was discovered that one member of the C/D box snoRNP complex, i.e., Nop1p, was similar in terms of sequence and structural motifs to methyltransferases [16]. While all C/D box snoRNAs contain a C box and a D box, most of them also contain two additional components: the C′ box and the D′ box. One or two antisense elements (10–21 nt in length) are present upstream of the D box and/or the D′ box. The sequence of these antisense elements is complementary to that of the target rRNAs. Therefore, the snoRNAs bind to target rRNAs through these antisense elements [17–21].

**Fig. 4:**
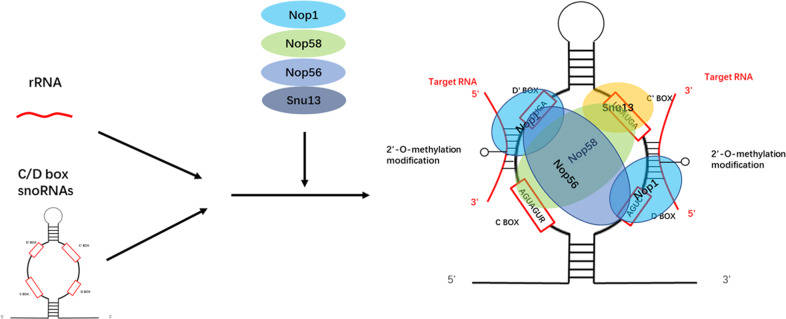
C/D box snoRNPs modifie the target RNA by 2′-O-methylation. SnoRNAs bind to target RNAs by antisense elements upstream of D box and/or D’ box. SnoRNAs form functional snoRNPs by binding to four core proteins, including Nop1p, Nop56p, Nop58p, and Snu13p. Among them, Nop1p transfers the methyl group on s-adenosine-methionine to the 2′- hydroxyl group of the target RNA ribose.

As a methyltransferase, Nop1p is a key component of snoRNPs [22]. It transfers the methyl group from SAM to the 2′-hydroxyl group of ribose molecules in the target RNA [23]. The introduction of the methyl group changes the spatial structure of the target RNA and increases its hydrophobicity, protecting the RNA molecule from nucleolytic attacks [24] (Fig. 4).

### Pseudouridylation

**Fig. 5:**
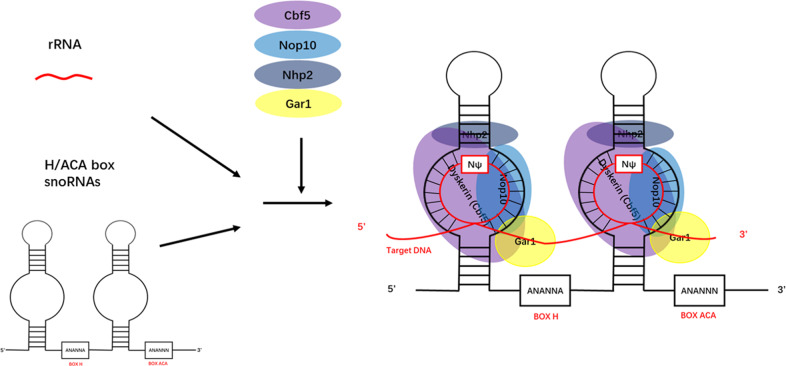
H/ACA box snoRNPs modifie the target RNA by Pseudouridylation. The H/ACA box snoRNAs form functional snoRNPs through binding to core proteins, including Cbf5p, Nop10p, Nhp2p and Gar1p. SnoRNPs bind to target RNA by their pseudouridylation pockets. In these pseudouridylation pockets, the target uridine in the target RNA is modified into pseudouridine.

Pseudouridylation is the most prevalent RNA modification and can be found in all species of cellular RNA [25, 26]. Pseudouridylation can maintain RNA stability and modulate ribosome synthesis. Further, it plays an important role in transforming nonsense codons into sense codons [27–29]. Cohn first found a new nucleoside in 1951, which was named pseudouridine after soon [30, 31]. Currently, there are two known modes of pseudouridylation: RNA-independent and RNA-dependent. RNA-independent pseudouridylation can be achieved by an enzyme called pseudouridine synthase, while RNA-dependent pseudouridylation requires H/ACA box snoRNPs [32, 33]. H/ACA box snoRNAs bind to target RNA and transform the target uridine into pseudouridine, increasing target specificity. This modification occurs in the pseudouridylation pockets of H/ACA box snoRNAs [34]. In addition, Cbf5p can also act as an independent pseudouridine synthase and modify transfer RNA (tRNA) substrates [35] (Fig. 5).

### N4-acetylcytidine (ac4C)

As a highly conserved RNA modification, ac4C can be found on both rRNAs, tRNAs, and mRNAs [36]. In 1978, Thomas et al. found that ac4C was present in the ribosomes of rats, chickens, and budding yeast [37]. Then, the acetyltransferases NAT10 and Kre33 were found to catalyze ac4C modifications in humans and yeast, respectively. NAT10 is an ATP-dependent RNA acetyltransferase and is necessary for cytokinesis and nucleologenesis [38, 39]. It has been reported that NAT10 can also regulate DNA damage responses and telomerase function [38, 39]. Eukaryotic 18 S rRNAs contain two acetylated cytidines, one in helix 34 and the other in helix 45. The former is crucial for translation fidelity, whereas the latter is part of the ribosome decoding site. With the help of snoRNAs, NAT10 can catalyze the formation of ac4C on rRNA [40, 41].

Sharma et al. found that Kre33 could bind to rRNAs and tRNAs in yeast. Moreover, they showed that Kre33 could also bind to two orphan snoRNAs, snR4 and snR45. Through quantitative reverse-phase high-performance liquid chromatography (RP-HPLC), they confirmed that low levels of snR45 were related to a decrease in 18 S rRNA acetylation [38]. Tyc et al. discovered that in vertebrates, the C/D box snoRNA U13 is complementary to the 3′ end of 18 S rRNA owing to the presence of two extended complementary base pair regions [42]. Meanwhile, Sharma et al. showed that residue 1842, which is present between these two regions of complementarity, corresponds to the acetylated residue C1773 in yeast. They hypothesized that the C/D box snoRNA U13 could be involved in 18 S rRNA acetylation. Using HPLC, they found that the acetylation levels of 18 S rRNA purified from HCT116 cells were reduced by half after the depletion of the C/D box snoRNA U13 for 72 h [38]. These findings strongly indicated that snoRNAs play a role in 18 S rRNA acetylation.

### Regulation of alternative splicing (AS)

AS is a process through which different combinations of splice sites are selected from a pre-messenger RNA (pre-mRNA) to produce variably spliced mRNAs [43]. In eukaryotes, AS significantly enriches the proteomic and transcriptomic output of the coding genome. In addition, AS is important for gene expression [44]. Multiple mRNA subtypes are generated from the same gene through AS in mammals. Due to their different coding capacities, stabilities, and translational efficiencies, these subtypes are translated into proteins with different structures and functions [45]. A primary RNA transcript contains exons, introns, and intervening sequences. Pre-mRNAs are converted to mature mRNAs through the removal of introns and the joining of spliced exons. This intron excision process is catalyzed by the spliceosome [42]. Moreover, AS allows the generation of mRNAs with different structures and functions, and therefore, different encoded proteins. It also influences intracellular localization, protein stability, enzymatic activity, and posttranslational modification of gene products [46]. Some studies show that numerous snoRNAs do not have binding site with rRNAs [47]. These indicate that snoRNAs could have functions beyond the modification of rRNA. Falaleeva et al. demonstrated that SNORD27 is present in nuclear components that lack FBL. The whole genome was searched for potential targets complementary to the SNORD27 sequence, and complementarity was identified between the alternatively spliced exons of the E2F7 gene and SNORD27. Further, low levels of SNORD27 were found to be associated with reduced levels of alternative exon skipping [48]. Moreover, Cavaillé et al. found that the orphan snoRNA SNORD115 plays a role in regulating the AS of serotonin receptor 2c (Htr2c) mRNA [12]. These findings indicate that snoRNAs may be involved in the regulation of AS.

### MicroRNA(miRNA)-like functions

miRNAs are short regulatory RNAs that can regulate post-transcriptional gene expression. Lai found that miRNAs inhibit translation by binding to specific sequences in the 3′-untranslated region, thereby performing regulatory functions [49].

Surprisingly, in 2008, Ender et al. used northern blotting and verified that small RNAs could be derived from the snoRNA ACA45. They also found that these ACA45-derived small RNAs had miRNA-like functions, through which they could target CDC2L6 [50]. Additionally, Ono et al. demonstrated that while the C/D box snoRNA HBII-180C contains a 2′-O-methylation site, it also has an M-box region, through which it can act as a miRNA and inhibit the mRNA and protein expression of target genes [51]. Hence, the literature suggests that some snoRNAs can play miRNA-like roles within cells.

## SnoRNAs in cancer

### SnoRNAs in lung cancer

Non-small-cell lung carcinoma (NSCLC) is a major contributor to cancer-related deaths. NSCLC accounts for more than 75% of all lung cancer cases, and patients are often diagnosed at an advanced stage, which considerably diminishes the probability of complete recovery [52, 53]. Despite the significant progress in treatments for lung cancer, the prognosis of NSCLC continues to remain dismal [54].

Cui et al. found that the levels of NOP10, a component of H/ACA box snoRNPs, are elevated in NSCLC and are related to poor outcomes. Additionally, they found that the reduction of pseudouridylation resulting from the knockout of SNORA7A, SNORA7B, and SNORA65 and the inhibition of NOP10 can decrease the proliferation, invasion, and migration of lung cancer cells. [55] According to Mourksi et al., low levels of SNORA80E can increase the rate of apoptosis and the cleavage of caspase-3 and PARP1 in lung cancer cells, while increased levels of this snoRNA is associated with reduced p53 levels. SNORA80E inhibits apoptosis through a p53-dependent pathway [56]. Zheng et al. showed that low levels of SNORD78 could also inhibit cell proliferation. This effect is likely related to the consequent increase in the proportion of G0/G1 cells. P21 and P16, which are G0/G1 arrest markers, are up-regulated after SNORD78 knockdown. Moreover, the low expression of SNORD78 also increases the proportion of Bax/Bcl-2-positive cells, thus promoting cell apoptosis. Overall, the results indicate that low levels of SNORD78 promote apoptosis and induce cell cycle arrest, thereby inhibiting cell proliferation [57]. In addition, Tang et al. demonstrated that SNORA71A could influence the cell cycle, cell migration, cell invasion, and the epithelial–mesenchymal transition (EMT) via the phosphorylation of MEK and ERK1/2 in MAPK signaling pathway [58]. Taken together, these findings suggest that snoRNAs are involved in the development of NSCLC.

### SnoRNAs in colorectal cancer (CRC)

CRC is a common type of cancer with the fourth-largest contributor to cancer mortality [59]. The treatments for CRC include surgery, chemotherapy, radiotherapy, and targeted therapy. However, despite the rapid development of therapeutic strategies against CRC, the prognosis of patients with CRC is still poor [60].

Owing to increasing research on snoRNAs, new evidence supporting the association between snoRNAs and CRC development has been uncovered. Liu et al. found that SNORD1C promotes the development of CRC by regulating β-catenin and TCF7 expression. High levels of SNORD1C are associated with a reduced five-year survival rate in CRC patients [61]. Another study showed that SNORA21 can promote CRC cell proliferation by regulating cancer-related pathways such as Hippo signaling pathway and Wnt signaling pathway and so on, and that high levels of SNORA21 is related to distant metastasis in CRC [62]. Fang’s group showed that in CRC, SNORD126 up-regulates FGFR2, thereby activating the PI3K-AKT pathway. The proteins downstream of this pathway include CREB, P27, MDM2, IKK, mTOR, p70S6K and GSK-3β. The overexpression of SNORD126 promotes the phosphorylation of GSK-3β and p70S6K, and promote the development of CRC via the PI3K-AKT signaling pathway [63]. SNORD12C/78 regulates the expression of target genes EIF4A3 and LAMC2 in a ZFAS1-dependent manner through NOP58-mediated 2’-O-methylation, promoting the development of CRC [64]. Hence, snoRNAs could be viable therapeutic targets for CRC.

### SnoRNAs in gastric cancer (GC)

GC, one of the most common malignant tumors, is the second-largest contributor to cancer-associated deaths in the world [65]. Like most tumors, the treatments of GC include surgery, radiotherapy, chemotherapy, and targeted therapy. Notably, early diagnosis is believed to improve the treatment outcomes and prognosis of GC significantly.

The study showed that in GC, SNORD105B could promote tumorigenesis by binding to ALDOA and thereby upregulating the expression of C-myc [66, 67]. In addition, Liu et al. demonstrated that the overexpression of SNORA21 was associated with increased lymph node metastasis and distant metastasis in GC [68]. These studies suggest that snoRNAs may play a significant role in the occurrence and development of GC.

### SnoRNAs in breast cancer (BC)

BC is the most common malignant tumor among women and the primary cause of cancer-related death in this group [69]. Hence, there is a great need to find effective prognostic biomarkers and therapeutic targets for BC.

Su et al. found that the levels of FBL were elevated in BC. FBL, a core protein of C/D box snoRNPs, was found to be important for the accumulation of snoRNAs and could affect Myc levels [70]. In turn, Myc also induced FBL expression. Meanwhile, low levels of FBL increased p53 activity, while its overexpression reduced the p53 response. Therefore, the findings indicated that snoRNAs could contribute to the development of BC by modulating the p53 response [71]. Another study found that snoRNA U50 mediates the methylation of C2848 in 28 S rRNA [72], suggesting that it may act as a tumor suppressor-like gene. Additionally, Dong et al. discovered that snoRNA U50, which inhibits cell colony formation, is frequently downregulated in BC [73]. Hence, snoRNA U50 may exert tumor-suppressive effects in BC. SNORD50A/B significantly enhances their interaction by forming a complex between the E3 ubiquitin ligase TRIM21 and its substrate GMPS, thereby promoting GMPS ubiquitination. Deletion of SNORD50A/B in p53 wild-type breast cancer cells releases GMPS and induces GMPS translocation into the nucleus, where GMPS can recruit USP7 and form a complex with p53, thereby reducing p53 ubiquitination, stabilizing p53 protein, and suppressing malignant phenotypes [74]. Kim et al. indicated that SNORA73A、SNORA73B and SNORA74A bound to PARP-1 to activate the catalytic activity of PARP-1 and mediated ADPRylation of DDX21, so as to promote cell proliferation in BC [75]. Moreover, Hu et al. found that in BC, SNORA71A promoted the binding of G3BP1-ROCK2 and increased the expression of ROCK2, promoting EMT process [76]. These evidences together support the association of snoRNAs with the occurrence and development of BC.

### SnoRNAs in Hepatocellular carcinoma (HCC)

HCC is common cancer with a high mortality rate [77]. Currently, therapies for HCC include surgery, radiotherapy, chemotherapy, and other comprehensive treatments, which extend life expectancy to a certain extent [78, 79]. However, owing to the unavailability of early screening markers, most patients with HCC are diagnosed in the advanced stage, and the lack of effective treatment leads to high rates of mortality [80].

Fang et al. found that orphan snoRNA SNORD126 promotes cell growth in HCC and binds to hnRNPK protein to up-regulate FGFR2, thus activating the PI3K-AKT pathway. Meanwhile, FGFR2 downregulation suppresses the growth of Huh-7 cells with high levels of SNORD126. Hence, SNORD126 appears to regulate HCC development via the PI3K-AKT pathway [81]. SnoU2_19 participates in the regulation of the Wnt/β-catenin signaling pathway by inducing the translocation of β-catenin between the cytoplasm and nucleus, thereby promoting the progression of hepatocellular carcinoma [82]. SNORD52 upregulated CDK1 by binding and enhancing the stability of CDK1 proteins to promote HCC tumorigenesis [83]. SNORD17 reduces p53 activation by anchoring nucleophosmin 1 and MYB-binding protein 1a in the nucleolus to drive HCC progression [84]. In addition, other studies have shown that the overexpression of SNORD105 can increase cell viability and motility in HCC [85] and SNORA42 can promote the development of HCC by inhibiting p53 signal pathways [86]. Further, SNORD113-1 suppresses tumorigenesis in HCC by regulating the transforming growth factor-β (TGF-β) and mitogen-activated protein kinase/extracellular signal-regulated kinase (MAPK-ERK) pathways [87]. These studies indicate that snoRNAs are involved in the development of HCC.

### SnoRNAs in ovarian cancer (OC)

Of all gynecological tumors, OC has the highest mortality rate [88]. Despite advancements in surgery and chemotherapeutics, the five‐year survival rate in women diagnosed with OC remains below 30% [89].

Zhang et al. found that SNORA72 influences cell stemness in OC via the Notch1/c-Myc pathway [90]. In addition, SNORD89 can affect cell proliferation, invasion, migration, and self-renewal ability in OC by regulating the Notch1/c-Myc pathway [91]. Therefore, snoRNAs may play a role in the development of OC.

### SnoRNAs in leukemia

Leukemia is classified into several types, including acute lymphoblastic leukemia, acute myeloid leukemia (AML), chronic lymphocytic leukemia, and chronic myelogenous leukemia [92]. The main treatment strategies for leukemia include chemotherapy and radiotherapy, although the former causes severe toxicity and adverse effects.

Valleron et al. discovered that SNORD112, SNORD113, and SNORD114 are ectopically expressed at the DLK1-DIO3 locus in acute promyelocytic leukemia. Their study showed that the variants of SNORD114-1 cause cell cycle arrest at G0/G1 and inhibit cell growth [93]. Another study by Pauli et al. demonstrated that the knockout of SNORD42A could reduce 2′-O-methylation levels of U116, causing ribosome activity and protein translation decreased. SNORD42A deficiency could inhibit cell proliferation and colony-forming ability in malignant cells [94]. All in all, snoRNAs may be related to the development of leukemia (Table 1).

**Table 1:** The mechanism and function of snoRNAs in several types of cancers.

Cancer	snoRNA name	Expression	Mechanism/Pathway/Target	Function	Reference
Lung cancer	SNORA7A	Upregulation	unknown	Promote the cell proliferation, invasion, and migration	[55]
	SNORA7B	Upregulation	unknown		
	SNORA65	Upregulation	unknown		
	SNORA80E	Upregulation	P53	Inhibit apoptosis and support stemness	[56]
	SNORD78	Upregulation	unknown	promoted the cell proliferation invasion and EMT process	[57]
	SNORA71A	Upregulation	MAPK/MEK/ERK	Promote the cell proliferation, invasion, and migration	[58]
	SNORA47	Upregulation	PI3K/Akt/EMT	Promote cell proliferation, migration, invasion and EMT process and inhibit apoptosis	[95]
	snoRNA U3	Upregulation	P53	Involve in pre-rRNA processing and required for in vitro and in vivo tumorigenesis	[96]
	snoRNA U8	Upregulation			
	SNORA42	Upregulation	P53	Promote cell proliferation, migration, invasion and xenograft growth in vivo	[97]
Colorectal cancer	SNORD1C	Upregulation	Wnt/β-catenin	Promote cell proliferation, migration, invasion and inhibit apoptosis and enhance cancer cell stemness	[61]
	SNORA21	Upregulation	Hippo signaling pathway and Wnt signaling pathway	Promote cell proliferation	[62]
	SNORD78	Upregulation	Regulate the expression of EIF4A3 and LAMC2 in a ZFAS1-dependent manner	Promote the development of CRC	[63]
	SNORD12C	Upregulation			
	SNORD126	Upregulation	FGFR2, PI3K-AKT/GSK-3β, p70S6K	Promote cell growth	[80]
	snoRNA U44	Upregulation	P53	Involve in p53-regulated cellular response to DNA damage	[98]
	snoRNA U47	Upregulation			
Gastric cancer	SNORD105B	Upregulation	ALDOA / C-myc	Promote cell proliferation, migration and invasion	[65, 66]
	SNORA21	Upregulation	unknown	Be associated with increased lymph node metastasis and distant metastasis	[67]
Breast cancer	snoRNA U50	Downregulation	Methylation of C2848 in 28 S rRNA	Inhibit cell colony formation	[71, 72]
	SNORD50A/B	Upregulation	TRIM21-GMPS/P53	Enhance malignant phenotypes in p53wt breast cancer cells, while inhibit malignant phenotypes in p53mt breast cancer cells	[73]
	SNORA73A/B	Upregulation	PARP-1/mediate ADPRylation of DDX21	Promote cell proliferation	[74]
	SNORA74A	Upregulation			
	SNORA71A	Upregulation	G3BP1/ROCK2	Promote EMT process	[75]
	SNORA71B	Upregulation	Unknown	Promote cell proliferation, migration, invasion and EMT process	[99]
	SNORA7B	Upregulation	unknown	Be related to poor prognosis for BC and promote cell proliferation, migration, invasion	[100]
Hepatocellular carcinoma	SNORD126	Upregulation	FGFR2, PI3K-AKT/GSK-3β, p70S6K	Promote cell growth	[80]
	snoRNA U2_19	Upregulation	Wnt/β-catenin	Promote cell proliferation and inhibit apoptosis	[81]
	SNORD52	Upregulation	CDK1	Be related to poor prognosis and promote cell proliferation	[82]
	SNORD17	Upregulation	P53	Promote the growth and tumorigenicity of HCC cells	[83]
	SNORD105	Upregulation	unknown	Increase cell viability and motility	[84]
	SNORA42	Upregulation	P53	Promote cell proliferation, migration, invasion and inhibit apoptosis	[85]
	SNORD113-1	Downregulation	TGF-β、MAPK-ERK pathways	Suppress HCC tumorigenesis	[86]
	ACA11	Upregulation	PI3K/AKT	Promote cell growth, migration, invasion and induce EMT process	[101]
	SNORA18L5	Upregulation	P53	Promote cell proliferation and inhibit apoptosis	[102]
	SNORD126	Upregulation	hnRNPK, FGFR2, PI3K-AKT	Promote HCC tumorigenesis in vitro and in vivo	[81]
	SNORA47	Upregulation	unknown	Promote cell proliferation, migration, invasion and inhibit apoptosis	[103]
	SNORD76	Upregulation	Wnt/β-catenin	Promote cell growth and induce EMT process	[104]
	SNORA42	Upregulation	P53	Promote cell proliferation, migration, invasion and inhibit apoptosis	[85]
	SNORA23	Downregulation	Impairing the 2′-O-ribose methylation of 28 S rRNA	Inhibit cell proliferation, migration and invasion	[105]
Ovarian cancer	SNORA72	Upregulation	Notch1/c-Myc	Induce the stemness of OC	[89]
	SNORD89	Upregulation	Notch1/c-Myc	Promote cell proliferation, migration and invasion	[90]
Leukemia	SNORD114-1	Ectopically expressed at the DLK1-DIO3	Rb/p16	Implicate in the G0/G1 to S phase transition	[92]
	SNORD42A	Upregulation in AML	Increase the 2′-O-methylation level of U116	Promote cell proliferation and colony-forming ability	[93]

## Conclusion

Previously, snoRNAs were thought only to be involved in 2′-O-methylation and pseudouridylation. However, with an increase in the number of studies, other functions of snoRNAs, including ac4C modification, AS regulation, and microRNA-like actions, have been discovered. Accumulating evidence indicates that the levels of snoRNAs are perturbed in malignant tissues. However, their specific roles in tumors have not been fully elucidated. In the existing studies, snoRNAs mainly bind to proteins, mRNAs, rRNAs, etc. directly or participate in protein regulatory pathways to regulate the modification and stability of proteins and RNAs, regulate protein expression and subcellular localization, and change the activity of proteins and protein complexes, thereby involved in tumorigenesis and cancer progression. According to the previous studies, 2’-O-methylation, pseudouracillation, ac4C modification, AS regulation, and microRNA-like effects also play key roles in tumorigenesis and cancer development. However, there are few reports that snoRNAs participate in tumor regulation by means of the above ways. So the questions whether and how snoRNAs use these pathways to participate in tumor regulation warrants further investigation. Such research could improve our understanding of the link between cancer and snoRNAs and bolster the use of snoRNAs as effective biomarkers and therapeutic targets for various cancers.

## Data Availability

The data used to support the findings of this study are available from the corresponding author upon request.
